# Enhanced proliferation and differentiation of mesenchymal stem cells by astaxanthin-encapsulated polymeric micelles

**DOI:** 10.1371/journal.pone.0216755

**Published:** 2019-05-20

**Authors:** Jun Zhang, Ching-An Peng

**Affiliations:** Department of Biological Engineering, University of Idaho, Moscow, ID, United States of America; Qatar University College of Health Sciences, QATAR

## Abstract

Astaxanthin is a highly potent antioxidant which can be extracted from *Haematococcus pluvialis* when cultivated and induced at high stress conditions. Due to astaxanthin’s hydrophobicity, methoxypolyethylene glycol-polycaprolactone (mPEG-PCL) copolymer was synthesized to form polymeric micelles for the encapsulation of astaxanthin. Astaxanthin-loaded polymeric micelles were then used to examine the effects on the proliferation and differentiation of human mesenchymal stem cells (MSCs). Dynamic light scattering (DLS) and Fourier transform infrared spectroscopy (FT-IR) confirmed astaxanthin was encapsulated into mPEG-PCL micelles. Astaxanthin loading and encapsulation efficiency, determined by UV/Vis spectroscopy, were 3.27% and 96.67%, respectively. After 48 h, a total of 87.31% of astaxanthin was released from the polymeric micelles. The drug release profile was better fit by the Michaelis-Menten type model than the power law model. The MSC culture results showed that culture medium supplemented with 0.5 μg/mL astaxanthin-encapsulated polymeric micelles led to a 26.3% increase in MSC proliferation over an 8-day culture period. MSC differentiation results showed that 20 ng/mL astaxanthin-encapsulated polymeric micelles enhanced adipogenesis, chondrogenesis, and osteogenesis of MSCs by 52%, 106%, and 182%, respectively.

## Introduction

Mesenchymal stem cell (MSC) has been widely used in tissue engineering and regenerative medicine due to its unique properties of self-renewal and multipotency [1]. Rapid *in vitro* expansion of MSCs is crucial for clinical applications [2]. Increased reactive oxygen species (ROS) levels have been shown to reduce the self-renewal ability and proliferation of MSCs [3]. Antioxidants are known to enhance *in vitro* proliferation of adipose-derived MSCs by regulation of cyclin-dependent kinase (CDK) and CDK inhibitor levels [4]. Proliferation of human and mouse bone marrow-derived MSCs have also been improved by supplementing culture medium with antioxidants such as ascorbic acid 2-phosphate, phenyl-α-tert-butylnitrone and N-acetyl-L-cysteine [5, 6]. Moreover, supplementation of ascorbic acid 2-phosphate also enhanced adipogenesis and osteogenesis of bone marrow-derived MSCs [6].

Astaxanthin is a lipid soluble, dark red carotenoid found in various aquatic animals [7]. It has been mainly used as a color additive in animal and fish feed, providing a pink to red-orange color to the flesh of aquatic animals [8]. Astaxanthin is also a very potent antioxidant, with an activity ten times higher than various carotenoids such as lutein, α-carotene and β-carotene [9]. The polyene chain and long conjugated double bonds are the reason for such strong antioxidant activity of astaxanthin [10]. Kim et al. reported that astaxanthin could improve the proliferation of neural stem cells; in addition, adipogenic and osteogenic differentiation of neural stem cells were also enhanced by astaxanthin [11]. In their study, astaxanthin was prepared as a stock solution in dimethyl sulfoxide (DMSO) due to its low solubility in water. However, using DMSO certainly is an issue when applying astaxanthin for *in vivo* applications [12]. Also, the effect of astaxanthin on chondrogenic differentiation remains unexplored. In this study, the effects of astaxanthin on mesodermal trilineage differentiation (i.e., adipogenesis, chondrogenesis and osteogenesis) of human MSCs were investigated together for the first time.

Astaxanthin production can be induced in microalgae *Haematococcus pluvialis*, one of the richest known sources of natural astaxanthin, when *H*. *pluvialis* is subjected to stressful conditions such as salinity, nitrogen deficiency, and light [13, 14]. In this study, the accumulation of astaxanthin was induced by the combination of high light stimulus and 15% CO_2_ aeration [15], and astaxanthin was extracted from *H*. *pluvialis* using mechanical disruption with DMSO [16]. After being extracted from *H*. *pluvialis*, a liquid-liquid extraction method was used to extract astaxanthin from DMSO to ether for obtaining astaxanthin powder by rotary evaporation.

Because poor water solubility of astaxanthin limits its applications, various carriers have been used to enhance the aqueous solubility of astaxanthin [17–21]. Polymeric micelle as an effective nanocarrier which has not been attempted for the encapsulation of astaxanthin. In this study, methoxypolyethylene glycol-polycaprolactone (mPEG-PCL), a biocompatible self-assembled diblock copolymer containing hydrophilic mPEG and hydrophobic PCL [22, 23], was synthesized to exam its entrapment capacity of astaxanthin in aqueous medium. The hydrophilic mPEG can protect the inner core from the aqueous environment, and the hydrophobic PCL is able to encapsulate poorly water-soluble astaxanthin. The chemical structure of astaxanthin-encapsulated mPEG-PCL micelles was characterized by Fourier transform infrared spectroscopy (FT-IR). The size of the micelles was measured by dynamic light scattering (DLS). UV/Vis absorbance spectrum was used to determine encapsulation efficiency (EE) and drug loading (DL). The release profile of astaxanthin from mPEG-PCL micelles was also obtained. The cultures of human bone marrow-derived MSCs were treated with various dosage of astaxanthin-encapsulated polymeric micelles to evaluate the effect of astaxanthin on the proliferation of MSCs. Our results showed that culture medium supplemented with 0.5 μg/mL astaxanthin-encapsulated polymeric micelles led to a notable increase in MSC proliferation over an 8-day culture period. Astaxanthin-encapsulated polymeric micelles were also supplemented in the media used for MSC mesodermal differentiation. As a result, media supplemented with 20 ng/mL astaxanthin-encapsulated polymeric micelles augmented mesodermal trilineage differentiation into osteoblasts, chondrocytes, and adipocytes.

## Materials and methods

### Materials

*Haematococcus pluvialis* (UTEX 2505) and MES-Volvox medium were purchased from the Culture Collection of Algae (UTEX, Austin, TX, USA). Astaxanthin standard, DMSO, acetone, methanol, 2,2'-azino-bis (3-ethylbenzthiazoline-6-sulphonic acid (ABTS), methoxypolyethylene glycol (mPEG; MW = 2,000), Tin(II) 2-ethylhexanoate (Sn(Oct)_2_), acetone, dichloromethane (DCM), phosphotungstic acid, rhodamine B, and penicillin-streptomycin were purchased from Sigma-Aldrich (St. Louis, MO, USA). Anhydrous ether, ammonium persulfate (APS), acetic acid, potassium hydroxide (KOH) and absolute methanol were purchased from J.T. Baker (Philipsburg, NJ, USA). ε-CL was purchased from Acros Organics (Geel, Belgium). Spectra/Por dialysis membrane tube (MW cutoff of 3.5 kD) was purchased from Spectrum Labs (Rancho Dominguez, CA, USA). Human bone marrow-derived mesenchymal stem cells were purchased from RoosterBio (Frederick, MD, USA). Minimum essential medium alpha medium (αMEM), L-glutamine and 0.25% trypsin/EDTA solution were purchased from Fisher Scientific (Waltham, MA, USA). Fetal bovine serum (FBS) was purchased from Gibco (Grand Island, NY, USA). AdipoLife DfKt-2 adipogenesis medium kit, OsteoLife complete osteogenesis medium, ChondroLife complete chondrogenesis medium, Oil Red O staining kit, 2% Alizarin Red staining kit, and Alcian Blue staining kit were all purchased from Lifeline Cell Technology (Frederick, MD, USA).

### Extraction of astaxanthin from *H*. *pluvialis* culture

Cultivation of *H*. *pluvialis* microalgal cells and the induction of astaxanthin were as reported previously [15]. Briefly, *H*. *pluvialis* were cultivated in MES-volvox medium in Erlenmeyer flasks under 80 μmol m^-2^s^-1^ light intensity (low light) for 8 days. At day 9, the light intensity was increased to 300 μmol m^-2^s^-1^ (high light) and 15% CO_2_ balanced with air was aerated into the culture to induce astaxanthin accumulation. The microalgal cells were cultivated for another two days at high light intensity and 15% CO_2_ stress conditions.

Extraction of astaxanthin from *H*. *pluvialis* has been previously described [24]. Briefly, the cells were harvested by centrifugation (4,500 rpm, 5 min), and re-suspended in 5 mL of 5% (w/v) KOH in 30% methanol. The cells were then heated in a 70°C water bath to destroy the chlorophyll. The cells were centrifuged again (4,500 rpm, 5 min) and then treated with 10 mL of DMSO with 5 drops of acetic acid. The cell suspensions were bath sonicated for 2 min three times with intervals of 30 s. The cells were then heated in the 70°C water bath for 5 min. The cells were heated repeatedly until they were completely white and finally centrifuged at 4,500 rpm for 5 min. The color of the cells was observed by Leica DMI3000 B microscope equipped with Leica EC3 digital color camera (Leica Microsystems, Wetzlar, Germany). The extracted astaxanthin in DMSO was stored at 4°C for further use.

### Extraction of astaxanthin from DMSO to ether

DMSO was mixed with water to determine the optimal DMSO:water ratio for astaxanthin extraction to ether. Three mL of astaxanthin solution was prepared in DMSO containing 1%, 3%, 5%, 10%, 20%, 30%, 40%, and 50% water, respectively. Three mL of ether was then added on top of all the prepared astaxanthin solutions. The mixture was fully mixed by vigorous shaking until the color of bottom layer (DMSO) turned from red to colorless. The UV/Vis absorbance spectrum of astaxanthin in DMSO/water mixture was recorded before and after extraction by a SpectraMax M2e microplate reader (Molecular Devices, Sunnyvale, CA, USA). The absorbance of astaxanthin in DMSO at 492 nm before extraction was taken as A_0_. The absorbance of astaxanthin in DMSO at 492 nm after extraction was taken as A_1_. The extraction efficiency was determined by the following equation:

$$astaxanthin\;extraction\;yield\;(\%)=\frac{A_{0}-A_{1}}{A_{0}}\times 100$$ (1)

An astaxanthin standard curve was constructed by making serial dilutions of astaxanthin standard solutions in DMSO. The concentrations of astaxanthin solutions were correlated with UV/Vis absorbance readings at 492 nm. Astaxanthin in ether was then dried by a rotary evaporator (IKA, RV 10, Staufen, Germany) at 40°C.

### Production of mPEG-PCL copolymer

The production of mPEG-PCL copolymer has been described by Sawdon et al [25]. Briefly, 50 mg of mPEG (molecular weight of 2,000) was mixed with 2.25 mL of ε-CL in the ultrasonication bath for 5 min at ambient temperature. Sn(Oct)_2_ (0.5 wt% of ε-CL) was added into the mixture, and the solution was transferred into a 3-necked round-bottom flask. The system was immersed in an oil bath at 140°C and continuously purged with nitrogen for 24 h. The crude product was allowed to cool to the ambient temperature, dissolved in DCM, and precipitated by treating the solution with cold methanol.

### Encapsulation of astaxanthin by mPEG-PCL polymeric micelles

Ten mg of mPEG-PCL was dissolved in 2 mL of acetone. An excess amount (5 mg) of astaxanthin was added into the solution and placed in an ultrasonication bath for 5 min. The mixture was added dropwise into 10 mL of DI water under ultrasonication. Acetone in the solution was removed by rotary evaporation at 60°C. The final micelle product was passed through a 0.45-μm filter. The amount of encapsulated astaxanthin was determined by UV/Vis spectroscopy and the astaxanthin standard curve. DL was calculated by the following equation:

$$DL=\frac{Amount\;of\;encapsulated\;astaxanthin}{Amount\;of\;polymer+Amount\;of\;encapsulated\;astaxanthin}\times 100\%$$ (2)

EE was determined by loading 0.3 mg of astaxanthin into 10 mg of mPEG-PCL micelles. The amount of encapsulated astaxanthin was determined by UV/Vis spectroscopy, and EE was calculated by the following equation:

$$EE=\frac{Amount\;of\;encapsulated\;astaxanthin}{Amount\;of\;original\;astaxanthin}\times 100\%$$ (3)

### ABTS scavenging activity

The ABTS scavenging activity of astaxanthin encapsulated micelle solution was evaluated using a previously describe method [26]. Briefly, 7 mM ABTS was mixed with equal volume of 2.4 mM APS, and the reaction mixture was allowed to react for 12 h at room temperature in the dark. The mixture was then diluted with deionized water, and the UV/Vis absorbance reading at 734 nm was taken as *K*_*0*_. 1 mL of ABTS solution was mixed with 1 mL of astaxanthin micelle solutions of different concentrations (10–100 μg/mL), and the reaction mixture was vortexed for 10 s. After 6 min, the UV/Vis absorbance reading at 734 nm was taken as *K*_*1*_. The ABTS radical scavenging activity was determined by the following equation:

$$ABTS\;scavenging\;activity\;(\%)=\frac{K_{0}-K_{1}}{K_{0}}\times 100$$ (4)

OD_734_ readings of astaxanthin-encapsulated polymeric micelles were measured to determine the ABTS scavenging activity.

### Characterization of astaxanthin-encapsulated mPEG-PCL polymeric micelles

The chemical structure of commercial astaxanthin, mPEG-PCL micelles, and astaxanthin-encapsulated mPEG-PCL micelles was characterized by FT-IR. FT-IR spectra were recorded by Spectrum One ATR/FTIR spectrometer (Perkin Elmer, Waltham, MA, USA). The particle size distribution of the mPEG-PCL micelles was determined by dynamic light scattering (DLS) using a Nano ZS Zetasizer (Malvern Panalytical, Westborough, MA, USA). The TEM samples were prepared by adding 10 μL of 1 mg/mL mPEG-PCL micelle solution onto a Formvar grid (Ted Pella, Redding, CA, USA) for 5 min and excess solution was wicked off. The samples were negatively stained with 10 μL phosphotungstic acid solution (2 wt%) for 10 s and excess solution was wicked off. Transmission electron microscopy (TEM) image of mPEG-PCL micelles was taken by JEM-4000FX (JEOL, Tokyo, Japan) at 80 kV.

### Astaxanthin release profile

Two mL astaxanthin-encapsulated mPEG-PCL micelle solution was placed in a Spectra/Por dialysis tube. The dialysis tube was then immersed in 500 mL 1X phosphate buffered saline (PBS) at ambient temperature. At specified time intervals, 100 μL of sample was removed from the dialysis tube and the absorbance at 492 nm was measured. The amount of astaxanthin released was quantified using the astaxanthin standard curve. All experiments were carried out in triplicate.

In order to identify the release mechanism of astaxanthin from mPEG-PCL micelles, the power law and the Michaelis-Menten model were used to fit the release profile. In the power law model, the *in vitro* drug release data were fit into the following equation:

$$\frac{M_{t}}{M_{\infty }}=at^{b}$$ (5)

where *M*_*t*_*/M*_*∞*_ is the fraction of astaxanthin release; *a* is a constant; *t* is the release time; *b* is the release exponent. Eq (5) is converted to a linear form, providing a linear fitting of the release data:

$$log\left(\frac{M_{t}}{M_{\infty }}\right)=log(a)+b\times log(t)$$ (6)

The release profile of astaxanthin from mPEG-PCL polymeric micelles was then modeled using the Michaelis-Menten model:

$$\frac{M_{t}}{M_{\infty }}=\frac{t}{K+t}$$ (7)

where *M*_*t*_*/M*_*∞*_ is the fraction of astaxanthin release; *K* is the Michaelis-Menten constant; *t* is the release time. Eq (7) is then converted to its linear form:

$$\frac{M_{\infty }}{M_{t}}=\frac{K}{t}+1$$ (8)

The coefficient of correlation *R*^*2*^ of the two models was compared to determine which model is more appropriate.

### Effect of astaxanthin-encapsulated mPEG-PCL on proliferation of MSCs

MSCs (passage 2) were cultured with αMEM supplemented with 16.5% FBS, 1% penicillin-streptomycin and 2 mM L-glutamine. MSCs were inoculated on 6-well plates at 5.0 x 10^4^ cells/cm^2^ and incubated at 37°C in a 5% CO_2_ incubator (Forma 310; Thermo Fisher Scientific, Waltham, MA, USA). Cellular uptake of mPEG-PCL polymeric micelles was evaluated by encapsulating 1 mg fluorescent rhodamine B. Free rhodamine B was removed by dialysis using Spectra/Por dialysis membrane tube (MW cutoff of 3.5 kD). EE and DL of rhodamine B by mPEG-PCL micelles was determined by measuring absorbance at 554 nm. MSCs were then treated with polymeric micelles loaded with rhodamine B for 4, 8, and 24 h. Phase contrast and fluorescent images were taken with a Leica DMi8 microscope equipped with Leica EC3 camera.

Astaxanthin-encapsulated mPEG-PCL micelles was prepared as describe in previous section. Astaxanthin-encapsulated micelles at 0, 0.25, 0.5 and 1 μg/mL were added separately to the MSC culture media for an 8-day period of cultivation; culture medium was replaced every 4 days. MSC culture with normal culture medium and MSC culture with the addition of mPEG-PCL micelles only were used as controls. The MSCs were harvested by treatment of 0.25% trypsin/EDTA, and the cell numbers were counted using a hemocytometer. The cell growth curve was determined to investigate the effect of astaxanthin-encapsulated mPEG-PCL micelles on the proliferation of MSCs.

### Effect of astaxanthin-encapsulated polymeric micelles on MSC differentiation

To evaluate the effect of astaxanthin-encapsulated mPEG-PCL micelles on MSC differentiation toward chondrogenic, adipogenic and osteogenic lineage, MSC differentiation were performed using differentiation medium kits according to manufacturer’s instructions (Lifeline Cell Technology). MSCs (passage 2) were inoculated in 6-well plates with αMEM supplemented with 16.5% FBS, 1% penicillin-streptomycin and 2 mM L-glutamine at the seeding density of 20,000 cells/cm^2^ for adipogenesis, 10,000 cells/cm^2^ for osteogenesis and chondrogenesis. After 24 h, culture medium was replaced with the corresponding differentiation medium kits supplemented with 20 ng/mL astaxanthin-encapsulated mPEG-PCL micelles. Untreated MSC differentiation media and MSC differentiation media supplemented with non-encapsulated mPEG-PCL micelles were used as controls. All media were changed every 3 days by replacing half of media from each well with fresh media. After 3 weeks of culture, chondrocytes were fixed with 4% paraformaldehyde and stained with Alcian Blue solution for overnight. Adipocytes were fixed with 4% paraformaldehyde stained with Oil Red O solution. Osteocytes were fixed with absolute methanol and stained with Alizarin red solution. Chondrogenic, adipogenic, and osteogenic differentiation were quantified by calculating blue, red, and black stained area respectively in at least 10 randomly selected microscopic fields using Image J (NIH, Bethesda, Maryland, USA).

### Statistical analysis

Statistical analyses were performed using Prism 8 software (Graphpad Software, San Diego, CA, USA). Data were presented as mean ± standard deviation from three independent experiments. For MSC proliferation and differentiation experiments, one-way analysis of variance, or ANOVA was performed followed by Tukey’s post-hoc analysis for multiple comparisons between different groups including unmodified control, mPEG-PCL micelles, and astaxanthin-encapsulated polymeric micelles of various concentrations. *p* < 0.05 was considered statistically significant.

## Results and discussion

### Cultivation of *H*. *pluvialis* and induction of astaxanthin

**Fig 1:**
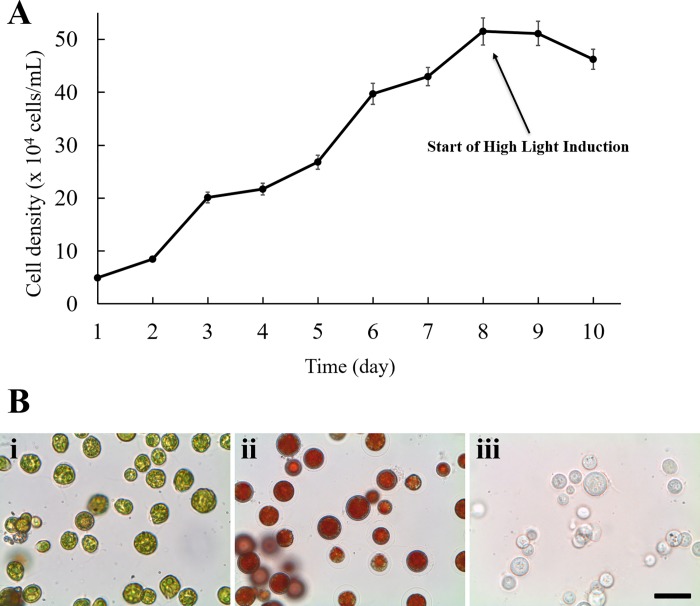
Growth kinetics and microscopic images of *H*. *pluvialis*. (A) Growth kinetics of *H*. *pluvialis* cultured at 80 μmol m^-2^s^-1^ light intensity (0–8 days) and then at 300 μmol m^-2^s^-1^ light intensity (9–10 days). Each value is expressed as the mean ± standard deviation (n = 3); (B) Microscopic images of *H*. *pluvialis* (i) at day 8 (before high light induction) (ii) day 10 (after high light induction) (iii) after astaxanthin has been extracted. Scale bar denotes 100 μm.

Fig 1A shows the growth kinetics of *H*. *pluvialis* for an 8-day culture period under low light conditions, then followed by a two-day astaxanthin induction period under high light and high CO_2_ stressful conditions. During the first 8 days, the cell density gradually increased from 10^5^ to 1.07 ×10^6^ cells/mL. However, after the inception of induction, the cell density slightly decreased from 1.07 ×10^6^ to 9.61 ×10^5^ cells/mL. Fig 1B showed the color change of the microalgal cells before and after the induction stage. The color of the algae remained green during the first cultivation stage. The color of the cells turned from green (Fig 1B-i) to red (Fig 1B-ii) after two days of induction under stress conditions. After astaxanthin was extracted, the *H*. *pluvialis* turned colorless (Fig 1B-iii).

### Extraction of astaxanthin from DMSO to ether

**Fig 2:**
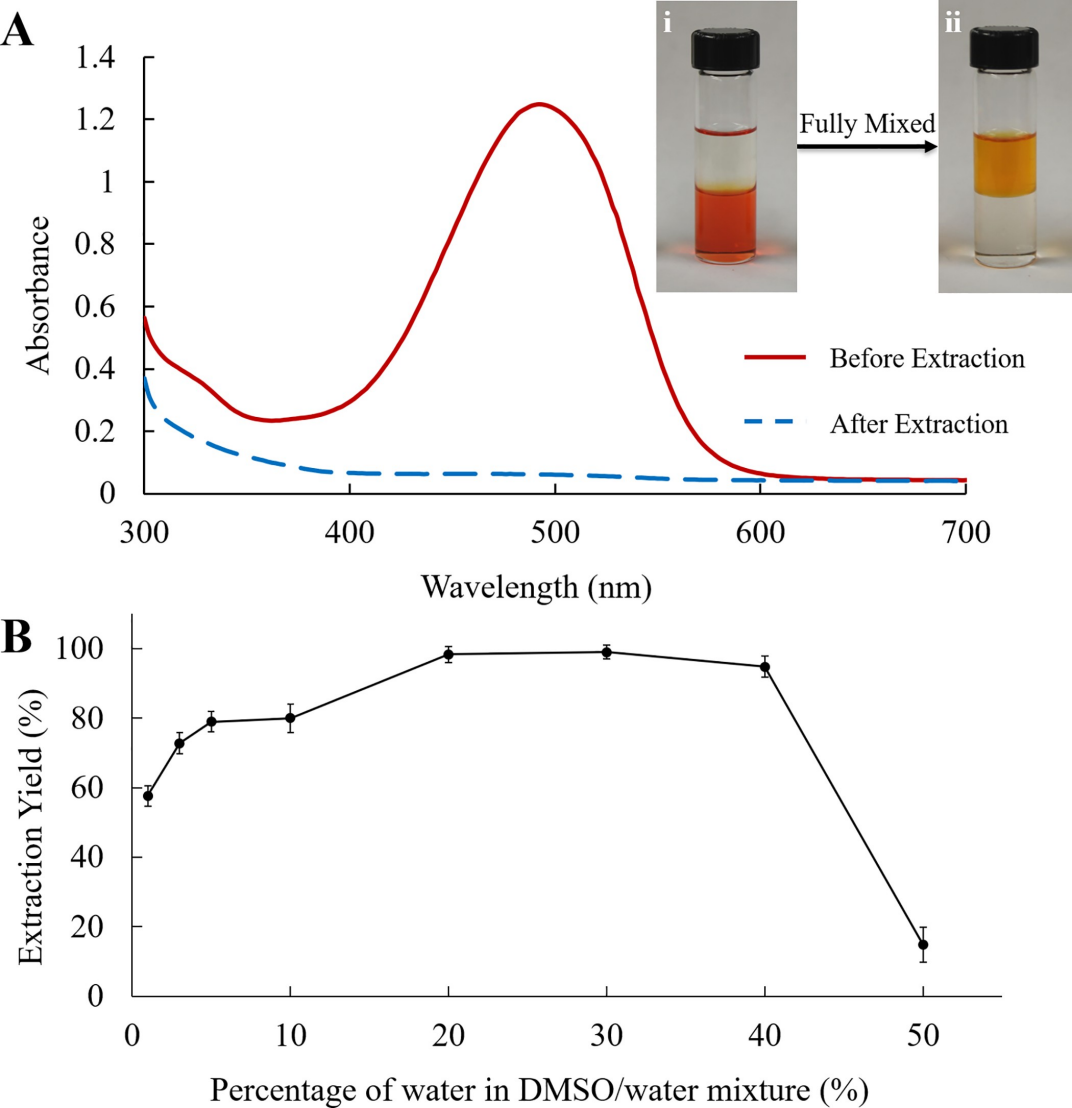
Liquid-liquid extraction of astaxanthin from DMSO to ether. (A) 1 mL of ether was added on top of 1 mL of astaxanthin in DMSO containing 20% water (i); the mixture was fully mixed (ii). The color of astaxanthin in DMSO turned from red to colorless. The ether phase turned from colorless to orange. The absorption spectra of astaxanthin stored in DMSO before and after extraction to ether were depicted. (B) The effect of water percentage (v/v) on the extraction yield of astaxanthin from DMSO/water mixture to ether. Each value is expressed as the mean ± standard deviation (n = 3).

Although DMSO is good for storing astaxanthin at 4°C, it is difficult to remove DMSO by rotary evaporation due to its high boiling point of 189°C. Astaxanthin stored in DMSO was extracted to ether by adding a layer of ether on top of the DMSO, followed by shaking the mixture. Astaxanthin extracts in DMSO were mixed with different volume of water to make astaxanthin solutions in DMSO/water. The volume ratio of DMSO/water to ether was fixed at 1:1 to find out the optimal water volume ratio for liquid-liquid extraction of astaxanthin. As shown in Fig 2A, the color of astaxanthin solution in DMSO/water was red (bottom layer), and the color of ether (top layer) was colorless before shaking. After shaking vigorously for about 10 s, the DMSO/water mixture turned from red to colorless, and the top layer turned from colorless to red/orange. This indicated most of the astaxanthin was successfully extracted from DMSO/water to ether layer. The absorbance spectra of astaxanthin in DMSO/water mixture were also measured before and after liquid-liquid extraction (Fig 2A). After extraction, the disappearance of absorbance peak at 492 nm also verified that astaxanthin was extracted from DMSO/water to ether.

The absorbance readings at 492 nm were used to determine the concentrations of astaxanthin solutions. The extraction yield was then calculated by the Eq (1) using the measured absorbance readings. Fig 2B depicts the extraction yield of astaxanthin plotted as a function of percentage of water in the DMSO/water mixture. About 98–99% of astaxanthin was extracted to ether when the volume of water was 20–40% (v/v). The extraction yield significantly dropped when the mixture contained 50% water (v/v). The optimal ratio of DMSO to water was 30%, giving an extraction yield of 99%.

### Encapsulation of astaxanthin by mPEG-PCL polymeric micelles

**Fig 3:**
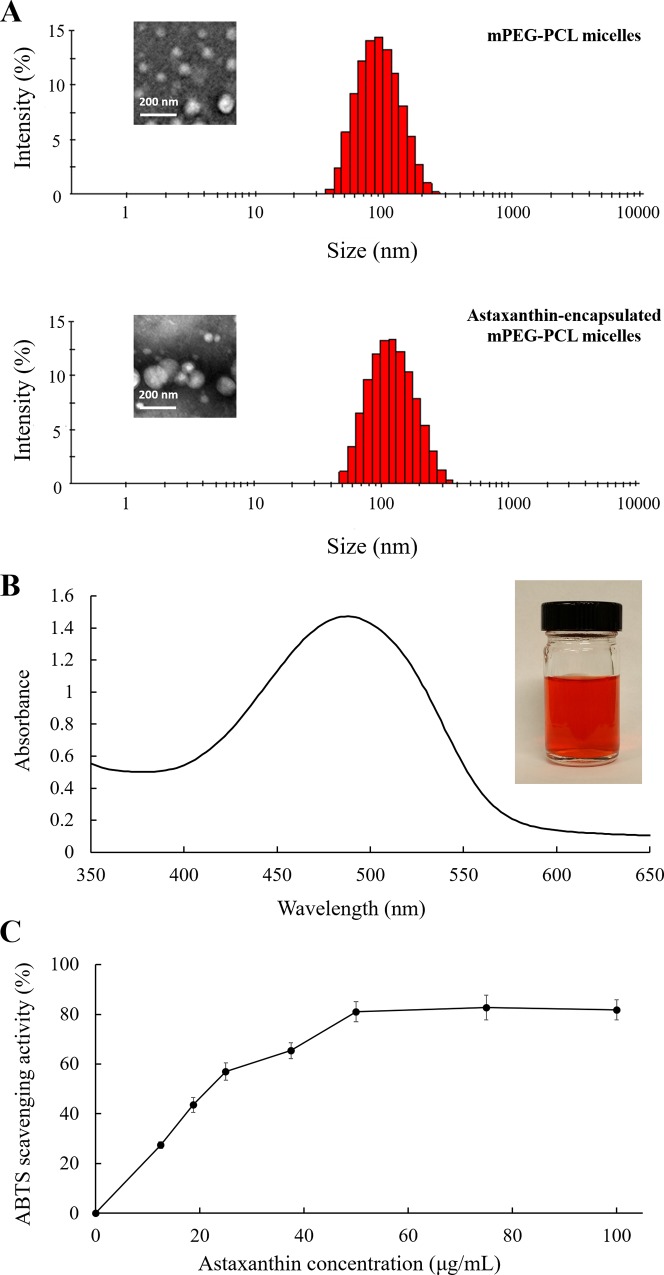
Characterization of astaxanthin encapsulated mPEG-PCL polymeric micelles. (A) Representative particle size distribution of mPEG-PCL polymeric micelles and astaxanthin-encapsulated mPEG-PCL polymeric micelles. The average size of mPEG-PCL polymeric micelles was 87.1 ± 9.1 nm; the average size of astaxanthin-encapsulated mPEG-PCL polymeric micelles was 112.3 ± 16.6 nm. Insets represent TEM images of corresponding micelles. Scale bar denotes 200 nm. (B) Absorption spectrum of astaxanthin-encapsulated mPEG-PCL polymeric micelles. The inserted image is the astaxanthin-encapsulated mPEG-PCL micelle solution. (C) The inhibition of absorbance at 734 nm as a function of astaxanthin concentration. Each value is expressed as the mean ± standard deviation (n = 3).

Astaxanthin was encapsulated in mPEG-PCL micelles to overcome the problem of its poor water-solubility. Amphiphilic mPEG-PCL copolymer was produced, and polymeric micelle solution was prepared by self-assembly process. Fig 3A shows the average particle size of mPEG-PCL micelles and astaxanthin-encapsulated mPEG-PCL micelles. The average particle size of mPEG-PCL micelles was 87.1 ± 9.1 nm in diameter. The average size of the astaxanthin-encapsulated mPEG-PCL polymeric micelles increased to 112.3 ± 16.6 nm which is 28.9% increment of hydrophobic core volume, confirming the inclusion of hydrophobic astaxanthin in the core of polymeric micelles. The morphology of mPEG-PCL micelles before and after astaxanthin encapsulation was shown in TEM images (Fig 3A). The TEM images showed that the size of mPEG-PCL micelles slightly increased after encapsulation, probably due to expansion of the PLC core after encapsulation of astaxanthin. The encapsulation of astaxanthin was further confirmed by observing the absorption peak at 492 nm of astaxanthin-encapsulated micelles as well as the red color of the micelle solution (Fig 3B).

### Astaxanthin loading and encapsulation efficiency by polymeric micelles

To determine drug loading percentage of astaxanthin by mPEG-PCL, 5 mg of astaxanthin was encapsulated by 10 mg of mPEG-PCL copolymer. The absorbance of astaxanthin-encapsulated micelles was examined, and the amount of astaxanthin was calculated by the astaxanthin standard curve. As a result, 0.338 mg was the maximum amount of astaxanthin to be encapsulated by 10 mg of mPEG-PCL copolymer in 2 mL of acetone. DL was 3.27%. The final concentration of astaxanthin-loaded mPEG-PCL micelle was 36.17 μg/mL, which is significantly higher than the reported 2 μg/mL concentration of astaxanthin-encapsulated Captisol (sulfobutyl ether β-cyclodextrin) [17]. The encapsulation efficiency was determined by loading 0.3 mg of astaxanthin into 10 mg of mPEG-PCL copolymer, and the astaxanthin amount was quantified by UV/Vis spectroscopy. As a result, 0.29 mg of astaxanthin remained in the final solution of polymeric micelles, and the encapsulation efficiency was 96.67%.

### ABTS radical scavenging

The antioxidant activity of astaxanthin solution was determined by the inhibition of absorbance of ABTS radical cations. Fig 3C depicts the inhibition of absorbance at 734 nm plotted as a function of astaxanthin concentrations. Our results showed that a 50 μg/mL astaxanthin solution inhibited the absorbance at 734 nm by 82.2±4.1%. The astaxanthin collected from 10 mL of 50 μg/mL astaxanthin extracts was re-dissolved in 10 mL DMSO. The re-dissolved astaxanthin solution inhibited the absorbance at 734 nm by 79.5±3.3%, which is similar to the antioxidant activity of above-mentioned astaxanthin extracted by DMSO/water mixture. This indicates that the activity of astaxanthin was not affected by the liquid-liquid extraction method. After encapsulation by mPEG-PCL, a 64.7 μg/mL astaxanthin-encapsulated polymeric micelle solution was able to inhibit the absorbance at 734 nm by 81.6±5.4%. This indicated that the ABTS scavenging activity of astaxanthin was retained after encapsulation by mPEG-PCL polymeric micelles.

### FT-IR spectrum of astaxanthin-encapsulated polymeric micelles

**Fig 4:**
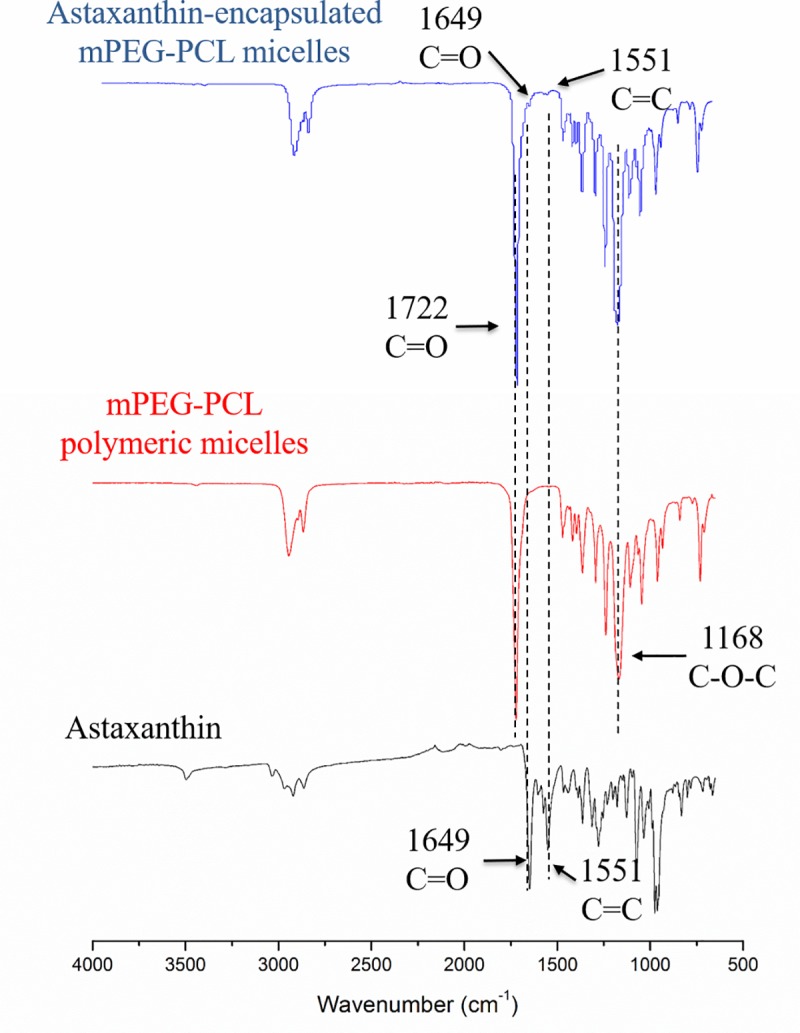
FT-IR spectra of astaxanthin, mPEG-PCL polymeric micelles, and astaxanthin-encapsulated mPEG-PCL polymeric micelles.

Fig 4 depicts the FT-IR spectra of astaxanthin, mPEG-PCL micelles, and astaxanthin-encapsulated mPEG-PCL micelles. In the spectrum of astaxanthin, the characteristic band appearing at 1649 cm^-1^ was due to the carbonyl group in astaxanthin. The peak at 1551 cm^-1^ was due to C = C in hexatomic ring [27]. The peak revealed at 963 cm^-1^ was due to C-H stretching. In the spectrum of mPEG-PCL micelles, the sharp peaks at 1722 cm^-1^ and 1168 cm^-1^ were due to the C = O and C-O-O stretching, respectively [28]. These two peaks indicated the formation of mPEG-PCL micelles was successful. The peak at 963 cm^-1^ for C-H stretching was also present in mPEG-PCL micelles. The sharp peak at 741 cm^-1^ was due to the aromatic C-H stretching in mPEG-PCL micelles. Comparing the spectrum of mPEG-PCL micelles and astaxanthin-encapsulated mPEG-PCL micelles, the peak appearing at 1551 cm^-1^ confirmed the encapsulation of astaxanthin. The characteristic peak for astaxanthin at 1551 cm^-1^ was weaker than that of commercial astaxanthin because the total amount of astaxanthin in the encapsulated micelles was low. The absorption peak for astaxanthin’s carbonyl group shifted from 1649 cm^-1^ to 1722 cm^-1^, probably because C = O stretching for mPEG-PCL micelles was much stronger than that of astaxanthin.

### Release of astaxanthin from polymeric micelles

**Fig 5:**
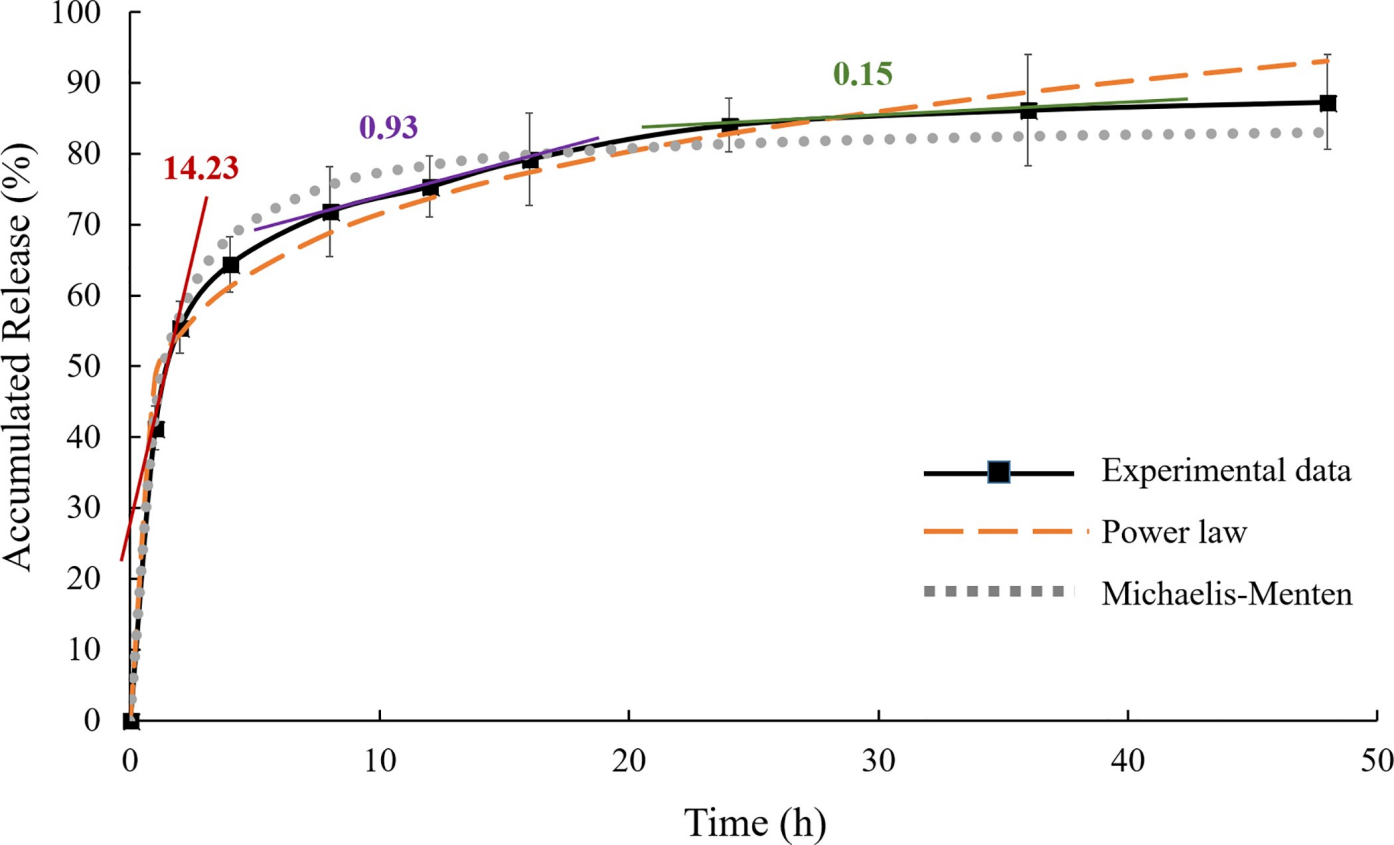
Astaxanthin release profile from mPEG-PCL micelles. Astaxanthin release profile from mPEG-PCL polymeric micelles in PBS at 25°C (mean ± standard deviation, n = 3). Plots generated from the power law and the Michaelis-Menten models were compared with the experimental data. Slopes were indicated on the in vitro release profile at different stages.

The release profile of astaxanthin from mPEG-PCL micelles in PBS at 25°C is shown in Fig 5. The release profile showed an initial burst release of astaxanthin for the first 8 h. In the initial burst release, the slope of the release profile was 14.23. Within 8 h, a total 71.85% of the encapsulated astaxanthin was released from mPEG-PCL micelles. After 8 h, the rate of astaxanthin release slowed down. A more sustained release pattern was observed from 8 to 48 h. The slope of the release profile decreased dramatically to 0.93 from 8 to 16 h. At the end of 48 h release, the cumulative release of astaxanthin from mPEG-PCL micelles was 87.31%. The slope decreased slight from 0.93 to 0.15 in the final stage.

In the power law model, the constant *a* was equal to 48.53, and the calculated release exponent *b* was equal to 0.17 for astaxanthin-encapsulated mPEG-PCL micelles. In a diffusion controlled drug release mechanism, the release exponent *b* of a sphere with Fickian diffusion is 0.43 [29]. The release exponent of astaxanthin-encapsulated mPEG-PCL micelles is less than 0.43, indicating that the release of astaxanthin is due to not only diffusion, but also degradation and collapse of the micelles [23, 30]. The release kinetics of astaxanthin from mPEG-PCL micelles was also modeled using the Michaelis-Menten model. The calculated Michaelis-Menten constant *K* was equal to 0.97. The Michaelis-Menten model fitted the experimental data better than the power law model. In the late stage of the generated plots, the Michaelis-Menten curve showed a smoother pattern than the power law curve, which is closer to the experimental data. Moreover, curve fitting using the Michaelis-Menten model resulted in a higher correlation coefficient (*R*^*2*^ = 0.98) than the power law model (*R*^*2*^ = 0.94).

### Effect of astaxanthin-encapsulated polymeric micelles on MSCs proliferation

**Fig 6:**
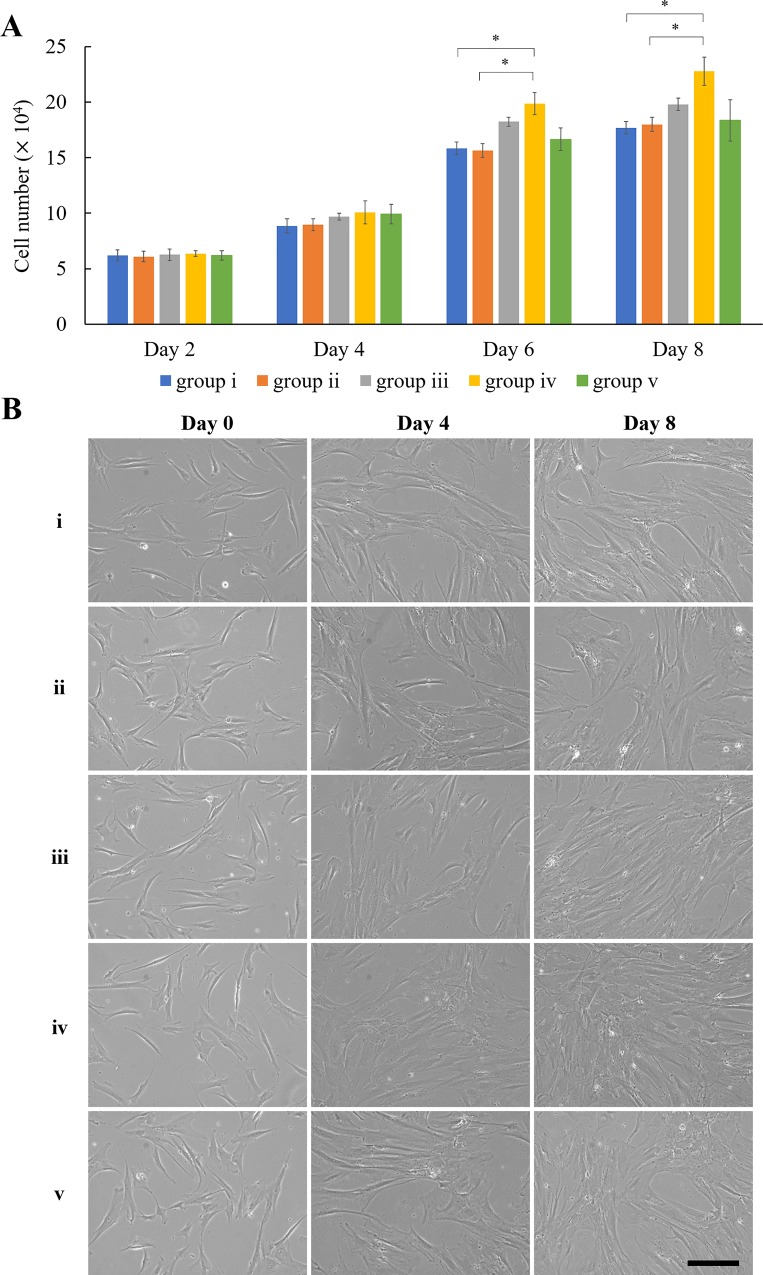
Effect of astaxanthin on proliferation of MSCs. (A) Proliferation of MSCs control (group i) and MSCs treated with 0, 0.25, 0.5 and 1 μg/mL astaxanthin-encapsulated mPEG-PCL micelles (group ii–v). (B) Microscopic phase contrast images of MSCs at day 0, 4, and 8, respectively. Scale bar denotes 100 μm. Data were presented as mean ± standard deviation. ANOVA with Tukey’s post-hoc analysis was performed (n = 3). * denotes *p* < 0.05.

Fig 6A reveals that, over the course of an 8-day culture, MSCs with mPEG-PCL micelles (no astaxanthin) had the same growth kinetics profile as the ones without the treatment of polymeric micelles. After 8-day cultivation, the control group (i) had 1.77 ×10^5^ cells/well, and non-encapsulated mPEG-PCL group (ii) had 1.79 ×10^5^ cells/well. However, the number of MSCs treated with astaxanthin-encapsulated polymeric micelles was higher than the control groups on the 8^th^ day post-inoculation: 1.98 ×10^5^ cells/well for group (iii) and 2.28 ×10^5^ cells/well for group (iv). Group (v) had 1.83 ×10^5^ cells/well, which was close to the cell numbers of group (i) and (ii). The addition of 0.5 μg/mL astaxanthin-encapsulated polymeric micelles (group iv) led to a 26.3% increase of cell number compared to the control group. However, further increase of astaxanthin concentration to 1 μg/mL led to less proliferation than group (iii). Fig 6B shows the MSCs treated with polymeric micelles containing 0.5 μg/mL astaxanthin reached confluency after 8 days of cultivation, yet cell confluency was not observed for those receiving 1.0 μg/mL astaxanthin-encapsulated polymeric micelles. This observation is in line with the cell numbers presented in Fig 6A. Judging from the morphology of MSCs for all study groups, there was no notable morphological change of cells indicating both polymeric micelles and astaxanthin had no effect on cell morphology. Kim et al. reported ~67% increase of proliferation of neural stem cells when supplementing cells with 10 ng/mL astaxanthin [11]. Similar decreasing trends of MSC proliferation with higher amount of antioxidant has been reported by Choi et al. [4]. They found that 250 μM ascorbic acid showed the highest MSC proliferation activity, but further increasing the concentration to 500 μM led to decrease of proliferation [6]. It is noteworthy that the efficacy of enhancing MSC proliferation is similar for both astaxanthin and ascorbic acid treatment, yet 0.5 μg/mL astaxanthin (approximately 0.84 μM) used in this study has much lower concentration than above-mentioned ascorbic acid. According to the release profile of astaxanthin given in Fig 5, ~87% of astaxanthin was released. Thus, the concentration of astaxanthin in culture medium was about 0.73 μM. This is probably due to the fact that the antioxidant potency of astaxanthin is much higher than that of ascorbic acid. According to our findings, it is surmised that astaxanthin reduced ROS level in MSC culture, thereby promoting the proliferation of MSCs.

**Fig 7:**
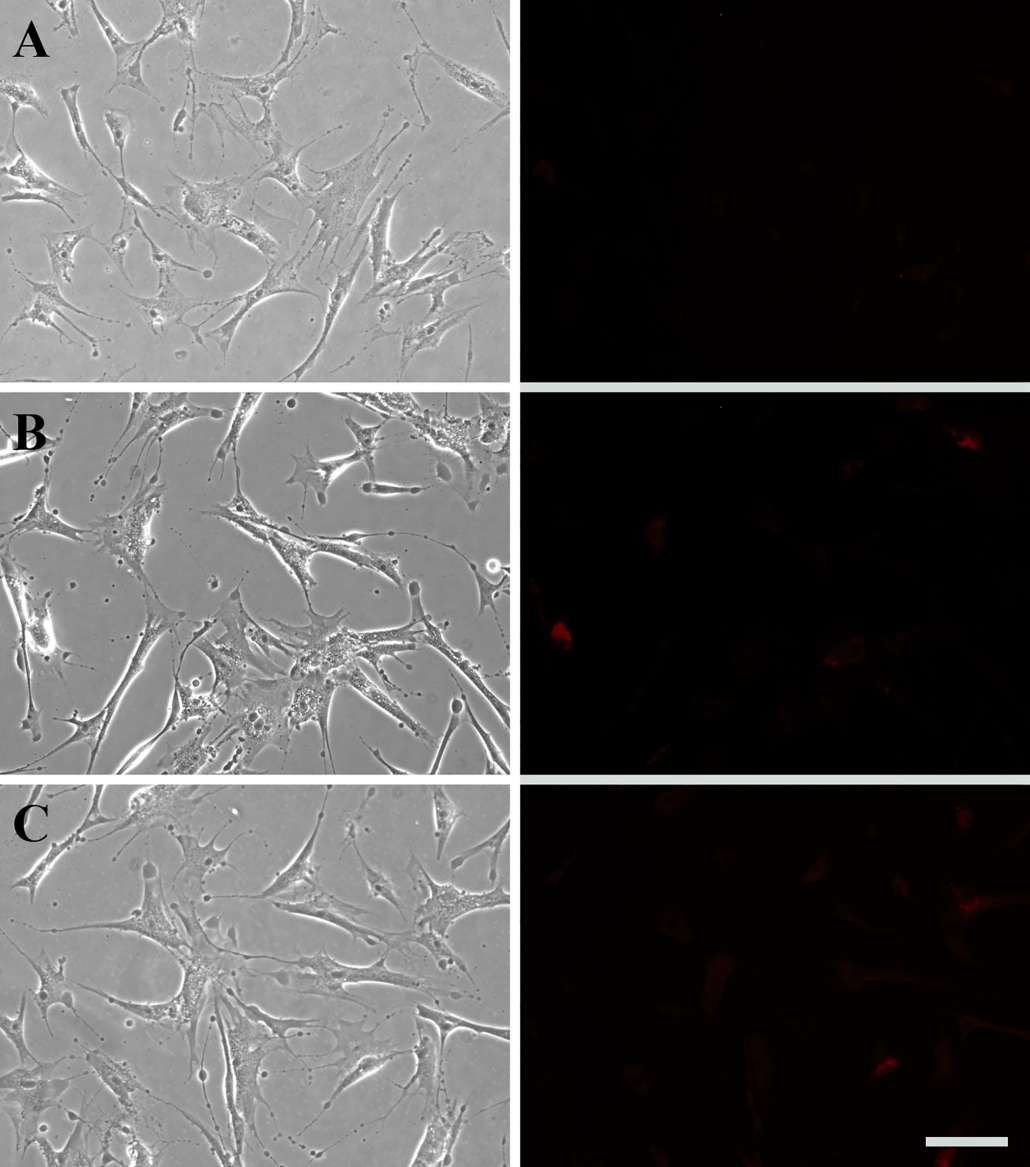
Cellular uptake of rhodamine B encapsulated mPEG-PCL micelles. Human MSCs were treated with rhodamine B encapsulated mPEG-PCL micelles for (A) 4 h, (B) 8 h, and (C) 24 h. Scale bar denotes 100 μm.

It should be noted that astaxanthin-encapsulated polymeric micelles could be ingested by MSCs, and therefore a portion of astaxanthin might be released intracellularly. To examine the endocytic process, MSCs were treated with rhodamine B encapsulated polymeric micelles. EE of rhodamine B by mPEG-PCL micelles was 62.5%. This was much lower than EE of astaxanthin (96.67%), probably due to less hydrophobicity of rhodamine B than astaxanthin. During encapsulation of rhodamine B, free rhodamine B in the aqueous solution was removed by dialysis, which was an extra step causing decreased EE. DL of rhodamine B by mPEG-PCL micelles was 3.03%, slightly lower than astaxanthin (3.27%). DL of rhodamine B was close to that of astaxanthin because the amount of mPEG-PCL polymer was significantly higher than the amount of encapsulated astaxanthin or rhodamine B, thereby only slight increasing in DL for astaxanthin. As shown in Fig 7, the fluorescent intensity of rhodamine B increased from 4 to 8 h post-treatment of polymeric micellar nanoparticles. The fluorescence intensity further increased from 8 to 24 h. This clearly showed that astaxanthin was gradually released from the polymeric micelles from extracellular to intracellular milieu, due to the internalization of micellular nanoparticles over the releasing period.

### The effect of astaxanthin-encapsulated mPEG-PCL on MSC differentiation

**Fig 8:**
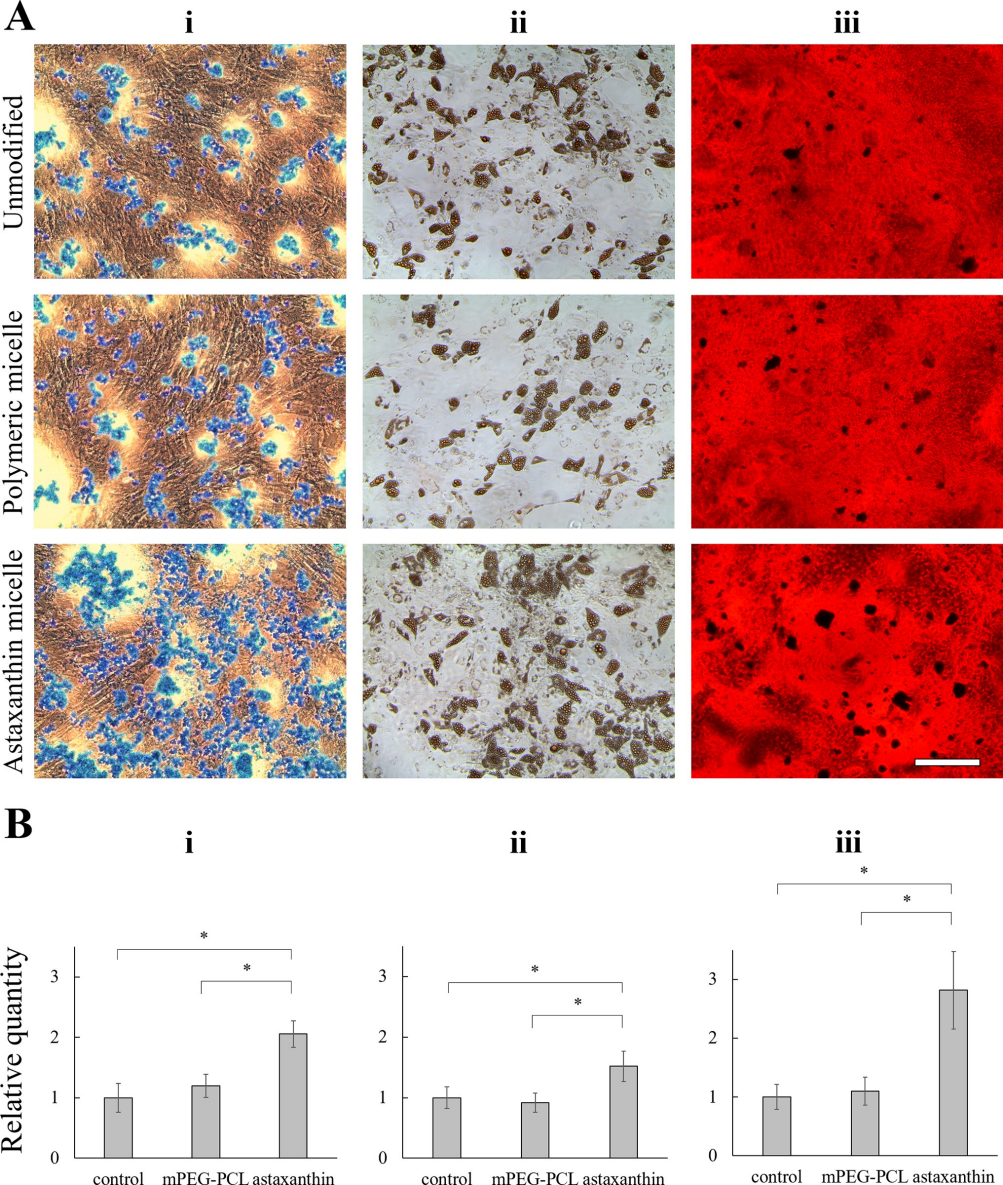
Effect of astaxanthin on differentiation of MSCs. (A) The effect of 20 ng/mL astaxanthin-encapsulated mPEG-PCL micelles on (i) chondrogenesis (ii) adipogenesis and (iii) osteogenesis of MSCs. MSCs treated with unmodified differentiation media and MSCs treated with non-encapsulated mPEG-PCL micelles were used as control groups. Chondrocytes were stained with Alcian Blue. Adipocytes were stained with Oil Red O. Osteocytes were stained with Alizarin Red. Scale bar denotes 100 μm. (B) Stained area of chondrocytes, adipocytes and osteocytes were quantified using Image J. Data was presented as mean ± standard deviation and normalized to the controls. Data were presented as mean ± standard deviation. ANOVA with Tukey’s post-hoc analysis was performed (n = 3). * denotes *p* < 0.05.

To determine the effect of astaxanthin on multipotency of MSCs, chondrogenic, adipogenic and osteogenic differentiation media supplemented with 20 ng/mL astaxanthin-encapsulated polymeric micelles were employed to induce MSC differentiation for 3 weeks. As shown in Fig 8A, chondrogenic, adipogenic and osteogenic differentiation of MSCs was achieved by using the media provided in the commercial kits without the addition of astaxanthin. MSCs treated with non-encapsulated mPEG-PCL micelles did not show any effect on MSC differentiation. MSCs treated with astaxanthin-supplemented differentiation media increased trilineage differentiation potency–chondrogenesis identified with sulfated proteoglycan stained by Alcian Blue (Fig 8A-i), adipogenesis demonstrated with lipid vacuole stained by Oil Red O (Fig 8A-ii), and osteogenesis visualized with calcium deposit stained by Alizarin Red (Fig 8A-iii). For chondrogenesis, measurements of the blue staining area indicated that astaxanthin led to 106% increase in chondrogenic differentiation of MSCs in 3 weeks compared to the control group (Fig 8B-i). For adipogenesis, astaxanthin-treated group showed moderate increase (52%) in formed red lipid vacuoles compared to the control group (Fig 8B-ii). For osteogenesis, astaxanthin-treated group showed 182% increase in black bone nodule formation compared to the control group (Fig 8B-iii).

The pleiotropic effect of astaxanthin on MSC differentiation into mesodermal lineages is clearly demonstrated. The significant increment on chondrogenic and osteogenic lineage is probably due to these two lineages share the same osteochondral progenitor cells [31], while adipogenesis has its own adipocyte progenitor. In fact, since chondrogenesis is so closely intertwined with osteogenesis, quite a few cytokines and chemicals used for the induction of chondrogenic differentiation are also implicated in osteogenic differentiation [32–34]. The overall enhancement of MSC trilineage differentiation induced by astaxanthin is probably because it acts as a potent scavenger against reactive oxygen species. It has been reported that supplementing antioxidant ascorbic acid is beneficial to the differentiation of bone marrow-derived MSCs into adipocytes, osteoblasts, and chondrocytes [6, 35]. Of note, the commercial differentiation media used in this study have already been supplemented with ascorbic acid. Apparently, astaxanthin had an add-on effect to augment MSC differentiation into mesodermal lineages. Since ascorbic acid can be rapidly oxidized resulting in short-time stability, the process involved in *in vitro* MSC differentiation is labor intensive (i.e., replacing differentiation media 2–3 times per week over 3–4 weeks). Using astaxanthin-encapsulated polymeric micelles could extend the stability of astaxanthin entrapped in the hydrophobic domain for sustained release, thereby decreasing the frequency of medium change.

## Conclusions

In this study, astaxanthin was extracted from *H*. *pluvialis* by mechanical disruption in DMSO. A liquid-liquid extraction method was used to extract astaxanthin from a DMSO/water mixture to ether. Astaxanthin was collected by rotary evaporation. mPEG-PCL polymeric micelles were used to encapsulate astaxanthin to enhance its aqueous solubility. The antioxidant activity of astaxanthin was retained after encapsulation by mPEG-PCL polymeric micelles. The *in vitro* release profile showed that astaxanthin was released from mPEG-PCL micelles in a sustained manner with an initial burst release. The addition of 0.5 μg/mL astaxanthin-encapsulated mPEG-PCL micelles to MSC cultures enhanced the proliferation of MSCs. Astaxanthin-encapsulated mPEG-PCL micelles with 20 ng/mL enhanced the chondrogenic, adipogenic and osteogenic differentiation of MSCs.

## Supporting information

S1 File PLOS one data set. (XLSX)
